# Supplementary material for: Mitochondrial lipidomes are tissue specific – low cholesterol contents relate to UCP1 activity
Source: Life Sci Alliance. 2024 Jun 6;7(8):e202402828. doi: 10.26508/lsa.202402828 (PMC11157264; doi:10.26508/lsa.202402828)
Supplement: Supplementary file 3 [file LSA-2024-02828_TableS1.docx]

**Table S1**: Known GPL/FC and FC contents of mammalian organelles

| **Organelle** | **GPL/FC** | **FC (%)** | **Reference** |
| --- | --- | --- | --- |
| Plasma membrane | 1.0-1.3 | 43.5-50.0 |  |
| ER | 6.7-14.3 | 6.5-13.0 |  |
| Golgi | 5.0-6.7 | 13.0-16.7 |  |
| Lysosomes | 2.0-2.6 | 27.5-32.9 |  |
| Peroxisomes | 12.5 | 7.4 |  |
| Nucleus | 4.5 | 18.0 |  |
| MAM | Not available | Not available | - |
