# Supplementary material for: Mitochondrial lipidomes are tissue specific – low cholesterol contents relate to UCP1 activity
Source: Life Sci Alliance. 2024 Jun 6;7(8):e202402828. doi: 10.26508/lsa.202402828 (PMC11157264; doi:10.26508/lsa.202402828)
Supplement: Supplementary file 4 [file LSA-2024-02828_TableS2.docx]

**Table S2**: The top 10 ranked lipid species most effectively classifying our samples after RF analysis of the training dataset

| **Rank** | **Lipid species** |
| --- | --- |
| 1 | PC 38:2 |
| 2 | FC |
| 3 | PE P 16:0/22:6 |
| 4 | PE P 18:0/22:6 |
| 5 | PS 38:3 |
| 6 | PE 40:7 |
| 7 | PC 34:2 |
| 8 | PE 34:2 |
| 9 | PE P 16:0/20:4 |
| 10 | PG 32:1 |
