# Supplementary material for: Mitochondrial lipidomes are tissue specific – low cholesterol contents relate to UCP1 activity
Source: Life Sci Alliance. 2024 Jun 6;7(8):e202402828. doi: 10.26508/lsa.202402828 (PMC11157264; doi:10.26508/lsa.202402828)
Supplement: Supplementary file 8 [file LSA-2024-02828_TableS3.docx]

**Table S3**: Composition of Control and HFD

| **Ingredients**  *Product No (Ssniff).* | **Control**  *S5745-E906* | **HFD**  *S5745-E920* |
| --- | --- | --- |
| Casein % | 24.000 | 24.000 |
| L-Cystine % | 0.200 | 0.200 |
| Corn starch % | 47.800 | 27.800 |
| Maltodextrin % | 5.600 | 5.600 |
| Sucrose % | 5.000 | 5.000 |
| Inulin ^1)^ % | 2.500 | 2.500 |
| Oat fiber ^2)^ % | 2.500 | 2.500 |
| Vitamin premix % | 1.200 | 1.200 |
| Mineral premix % | 6.000 | 6.000 |
| Choline Cl % | 0.200 | 0.200 |
| Palm oil % | —— | 20.000 |
| Soybean oil % | 5.000 | 5.000 |
| **Proximate contents** |  |  |
| Crude protein % | 21.1 | 21.1 |
| Crude fat % | 5.1 | 25.1 |
| Crude fiber ^1), 2)^  % | 4.8 | 4.8 |
| NDF ^1), 2)^ % | 2.3 | 2.3 |
| Soluble fiber ^1), 2)^ % | 2.1 | 2.1 |
| Total dietary fiber ^1), 2)^ % | 4.5 | 4.5 |
| Crude ash % | 5.4 | 5.4 |
| Starch % | 45.9 | 26.7 |
| Dextrin % | 5.5 | 5.5 |
| Sugar % | 6.2 | 6.2 |
| Energy (Atwater)^3)^ MJ/kg  Protein kcal%  Fat kcal%  Carbohydrate kcal% | 15.4  23  13  64 | 19.7  18  48  34 |

^1)^ Calculated with 94 % crude fiber, 90 % total dietary fiber and 85 % soluble fiber

^2)^ Calculated with 96 % crude fiber, 90 % total dietary fiber and 89 % insoluble fiber

^3)^ Physiological fuel value
